# Biological Anomalies around the 2009 L’Aquila Earthquake

**DOI:** 10.3390/ani3030693

**Published:** 2013-08-06

**Authors:** Cristiano Fidani

**Affiliations:** Central Italy Electromagnetic Network, 63847 San Procolo, Fermo, Italy; E-Mail: c.fidani@virgilio.it; Tel.: +39-0753-4060; Fax: +39-0753-4036

**Keywords:** plants, animals, humans, earthquakes, electric signals, positive holes, gas leaks

## Abstract

**Simple Summary:**

Earthquakes have been seldom associated with reported non-seismic phenomena observed weeks before and after shocks. Non-seismic phenomena are characterized by radio disturbances and light emissions as well as degassing of vast areas near the epicenter with chemical alterations of shallow geospheres (aquifers, soils) and the troposphere. Many animals are sensitive to even the weakest changes in the environment, typically responding with behavioral and physiological changes. A specific questionnaire was developed to collect data on these changes around the time of the 2009 L’Aquila earthquake.

**Abstract:**

The April 6, 2009 L’Aquila earthquake was the strongest seismic event to occur in Italy over the last thirty years with a magnitude of M = 6.3. Around the time of the seismic swarm many instruments were operating in Central Italy, even if not dedicated to biological effects associated with the stress field variations, including seismicity. Testimonies were collected using a specific questionnaire immediately after the main shock, including data on earthquake lights, gas leaks, human diseases, and irregular animal behavior. The questionnaire was made up of a sequence of arguments, based upon past historical earthquake observations and compiled over seven months after the main shock. Data on animal behavior, before, during and after the main shocks, were analyzed in space/time distributions with respect to the epicenter area, evidencing the specific responses of different animals. Several instances of strange animal behavior were observed which could causally support the hypotheses that they were induced by the physical presence of gas, electric charges and electromagnetic waves in atmosphere. The aim of this study was to order the biological observations and thereby allow future work to determine whether these observations were influenced by geophysical parameters.

## 1. Introduction

The April 6, 2009, L’Aquila earthquake struck the Abruzzo Region with a magnitude M = 6.3 at 3:32 LT, causing 307 deaths and considerable damage to structures over an area of approximately 600 km^2^, including the urban center of L’Aquila and several villages in the mid-Aterno Valley. It was the strongest event in a sequence of seismic events that had started a few months earlier. In fact, local seismic activity began to increase in October 2008 [1]. The most significant foreshocks occurred on March 30 (M = 4.4) at 15:38 LT, April 5 (M = 4.2) at 22:48 LT and April 6 (M = 3.9) at 00:39 LT [2]. The main shock was followed by seven aftershocks in the first week; the moment magnitude was M_w_ ≥ 5, with the two strongest occurring on April 7 (M = 5.6) at 19:47 LT and on April 9 (M = 5.4), at 02:52 LT [3]. The affected area was within the central section of the Apennines, the mountain chain, which traverses most of the Italian peninsula. The L’Aquila basin is a vast intra-Appennine tectonic basin elongated in a NW-SE direction, parallel to many active normal faults, surrounded by the high peaks of the Gran Sasso and the Velino-Sirente mountains chains. These latter two host national and regional parks (http://www.parks.it/regione.abruzzo/Eindex.php), respectively, where a lot of animal species live. The Appennines in Central Italy, near the epicenter, are made up of many protected areas including: The National Park of Abruzzo, The Regional Park of Majella, The Regional Green Areas of The Zompo lo Schioppo and The Twin Mountains, as well as The National Park of Sibillini (http://www.parks.it/parco.nazionale.monti.sibillini/Eindex.php). All of these areas are part of a future project called Appennino Parco d’Europa (http://www.convenzionedegliappennini.it/home.php?lang=2), which will include the areas between the above mentioned parks. This project will protect the flora and fauna richness of this territory. Furthermore, in Central Italy, there is a diffused custom among the citizens to keep and breed farm animals, which seldom live in complete fusion with the natural environment. Protected areas and farms are constantly monitored by foresters, provincial rangers [4] and breeders [5], which are the persons who administer questionnaires and/or possible references for setting up instruments. Given the above, it is believed that such sites are optimal for monitoring animal behavior.

Past observations of animal behavior before, during and after earthquakes, have been reported since the early BC centuries in Central Italy [6]. However, a valid scientific approach requires being able to reproduce an observation via an experiment. One of the first to suggest so was the Jesuit Priest Alessandro Serpieri at the University of Urbino, Marche, Italy, at the end of the XIX century. He suggested to Prof. Antonini to carry out observational experiments on the nature of abnormal snake behavior before an earthquake [7]. The first reported observation dealt with a laboratory slowworm (*Anguis fragilis*) that woke up before the end of lethargy and hissed. This was observed by Antonini slightly before the great earthquake of March 12, 1873, M = 5.9, in San Ginesio, Central Italy [8]. After this quake, the laboratory snake fell back into lethargy for several days. Antonini sought to reawaken the snake by using electric currents. Antonini stated that: *“I took the little box where I kept the snake imprisoned, and positioned the box on a table without the cover and I began to rock the table. I sent him from side to side, but the snake did not awaken or hiss. Therefore, I positioned the box on a wooden floor and I sat in the company of my boys, wobbling the floor. However, this did not awaken the snake. Finally, I placed the box on a large table, and I hit it suddenly with violent shocks, only then did the snake begin to move a little. It moved its head from left to right, but did not awaken. I repeated this for a few days. But always obtaining the same results. Eventually, I got the idea to perform another test. I used a small electric generator at induction: charged its battery with bi-sulphate of mercury, and touched the slowworm with the two poles, one on the back and the other on the tail. Then the poor animal shook violently and sent a puff and lengthened its forked tongue. At this point, I thought of killing it. I repeated the test, but I knew that the poor animal was in great pain, so I stopped; wanting to stop my other special observations”*. This experiment was suggested by Serpieri who suspected that *“...the unknown cause of the strange animal behavior had an electric nature, and animals were more excited than us...”* [7]. This hypothesis was inspired by the observation of atmospheric electricity during the same earthquake by Antonini in Montefortino [9].

A valid scientific approach also requires being able to systematically collect data. To this regard, in 1887, Mercalli and Taramelli composed a questionnaire, on macroscopic effects, which was compiled by more than one thousand witnesses [10], after the February 23, 1887, M = 6.5, Liguria earthquake [11]. The authors of the questionnaire had believed that biological anomalies could be useful in understanding the full earthquake process. The survey was carried out over the territory around the seismic epicenter. Mercalli concluded: *“In more than 130 locations, as is known to us, the witnesses were taken by the state of anxiety or fear on the part of pets. The animals let out unusual cries, exhibited restlessness: the fluttering of birds, that attempted to escape to the outdoors etc.., generally, a few minutes before the earthquake”*. Influenced by the Mercalli and Taramelli questionnaire, other authors in Italy have compiled their own surveys, specifically for the strong earthquakes occurring in the Friuli Region in Northern Italy in 1976 [6,12]. These surveys revealed that the animal behavior around the seismic events was similar to that recorded in 1887. One year prior to this, Chinese researchers had predicted a strong seismic event [13] after carrying out a survey on several geophysical parameters as well as animal behavior, even if in a skeptical way. It is estimated that this prediction saved thousands of lives in the region of Haicheng.

This study was also based upon a questionnaire prepared right after the L’Aquila earthquake [14] and given to 1,200 residents. Halfway between collecting and reproducing experiments there is a necessary intermediate scientific step for preparing the data. The purpose here is to present reports of phenomena noticed, giving them an order with respect to space, time and type. A great number of interviews were made face-to-face, and based on this, generally very much information was collected. Thus, the information must be treated gradually, ordering it more specifically to make correct sense and understanding of it. A first ordering of data was the major scope of the present work, taking into account not very precise times, spaces and kinds of biological life. Thus, the analysis was purposely chosen to be not as rigorous as in a recently reported work [15]. Future works will be more rigorous regarding analysis as in the case of a previous paper [16], following [14]. Being so, future publications will take advantage of this present work, which created a database with essential characters. It should be of practical use to better decide grouping of data for statistical analysis. However, it must be remembered that many reports may be psychological in origin or inaccurately recalled, or merely presented as co-incidental events, which would have happened anyway and not related to seismic activity. Section 2 below describes the methods used. Section 3 discusses the data while Section 4 deals with the conclusions.

## 2. Experimental Methods

Most of the collected data concerns 249 cases of luminous phenomena, which were for the most part observed during the coseismic phase of the main shock [14,16]. The remaining data, concerns reports of fluid emissions, chasms, thermal releases, excessive meteorological phenomena, unusual sounds, radio and telecommunication disturbances, changes in human body and changes in animal behavior [17]. The latter two, being the largest group with more than 500 cases, are the subjects of this analysis. A questionnaire was compiled to collect information on public perception [18] of these phenomena, for which no instruments were active at the time and at the site of the L’Aquila earthquake. The questions were inspired by previous works [12,19,20,21,22,23,24], and the questionnaire can be downloaded from the supplementary material of a previous paper [14] at http://www.nat-hazards-earth-syst-sci.net/10/967/2010/nhess-10-967-2010-supplement.pdf. The peculiar scheme of this data collection was addressed to: (1) instruct the general public on the existence of these earthquake-associated phenomena; (2) to propose new hypotheses on earthquake processes; and (3) suggest new devices for systematic recording of these phenomena. This line of research was proposed for the first time by Fidani [14] following a long period of reading and summarizing historical papers [25]. At the time of the interviews, more than 50% of the surveyed knew something about animal behavior during earthquakes. However, at the time of the main shock most people failed to connect the strange animal behavior with a possible future seismic event.

The survey began on 11 April 2009, five days after the main shock, in the Amatrice area (Lazio region), about 35 km north of L’Aquila, then moved onto the epicentral area through: Campotosto, Santa Vittoria, Montereale, Capitignano, San Giovanni, Marana, Cagnano Amiterno, Barete, Pizzoli, Arischia, San Vittorino, Preturo, Cesi, Scoppito, Civitatomassa, Sassa, Madonna della Strada, Pagliare, Foce, Colle Santa Maria, Tornimparte, Borgorosa, Massa D’albe, Rosciolo, Lucoli, Santa Ruffina, Roio Poggio, Roio Piano, Roio Colle, Genzano, Coppito, Cansatessa, Pettino, L’Aquila, Collebringioni, Aragno, Assergi, Camarda, Filetto, Paganica, Tempera, Gignano, Pianol, Bazzano, Monticchio, Onna, Vallesindola, Bagno, Civita di Bagno, Cavallette, San Felice d’Ocre, Fossa, Fonticchio, San Gregorio, Poggio Picenze, Barisciano, San Demetrio nei Vestini, Villa Sant’Angelo, Prata d’Ansidonia, Castelnuovo, Tussia, San Pio delle Camere, San Nicastro, Caporciano, Rocca Preturo and other smaller villages. Data were collected from 179 camps set up by the Italian Civil Protection Agency [26]. Testimonials were also collected from public places such as schools, malls, hospitals, coffee shops and social clubs. Furthermore, personnel at public and private institutions including police officers, firemen and forest rangers were interviewed. Interviews began from the least hit area, The Rieti Province, to the most hit area, The L’Aquila Province. The questionnaire started with questions of a general nature and led up to more specific ones; related questions were grouped which allowed for a smooth transition from one group to the next [27]. The question response format included a combination of both open questions to describe the observations and closed questions to obtain associated fact as well as to test the consistency of responses of each testimony. Questions dealt with demographic classification, knowledge and perception on the part of witnesses. Classification was limited to place of residence and the name of the witness, so they could be contacted for further questions if necessary [18].

The questionnaire was administered in a face-to-face mode, which reduced witness conditioning and allowed as much information as possible to be collected [18,28]. Telephone and e-mail interviews were done when witnesses could not be reached physically. The interviewed areas were principally civil protection camps where people gathered for meals. The manner of interviewing was carried out on an individual basis and in groups of up to twenty people. When a testimonial was collected but made by a third party, a search was performed to track down the original witness. Then, when possible, during the next trip to the zone, the original witness was interviewed or contacted by phone or e-mail. Non-probability techniques were used to select the sample: accidental, purposive or snowball [18]. These were principally used because of the social difficulties and the emergency housing situation. Therefore, statistical generalizations could not be made. However, this was not the aims of this work. About 1,200 interviews were carried out, but about 25% more people reported that they did not see anything and did not fill out a questionnaire. Less than 5% reported to have not noticed anything, while more than 50% reported multiple observations [14], which were later updated [16]. After revising biological anomalies, a total of 1,311 macroscopic anomalies were reported. A summary of the observational types is given in Table 1.

**Table 1:** Types of observations with respect to the distance from L’Aquila.

Type of observational	< 20 km (#)	> 20 km (#)	Total (#)
Earthquake lights	237	12	249
Radio-telecommunications	68	16	84
Unusual sounds	55	14	69
Unusual fluid emissions	144	18	162
Soil deformation	25	5	30
Unusual meteorology	140	26	166
Biological anomalies	467	84	551
All	1,136	175	1,311

Unusual animal behavior was reported far from the epicenter, up to 70 km [29], regarding several different species [30]. Most of the unusual animal behaviors were reported to appear minutes before the seismic events. Dogs were reported to have been barking by nearly all of the interviewed, seconds before the main shock. However, only the observations that were accompanied by precise descriptions of unusual behavior were included in this study and listed in Table 1. No audio recordings were collected in connection with the strange animal behavior. Other instrumental observations were made near the locations and at the time of strange animal behavior, such as temperature, wind speed, rainfall, water flux and magnetic field. Flora observations were also included in this study.

## 3. Results and Discussion

### 3.1. Plants

The survey reported 49 observations of abnormal growth patterns for: vegetables, fruit, yellow-green pollen clouds, mushrooms and lake algae.

#### 3.1.1. Pollen

One pollen observation was made weeks before the main shock at the Tempera cemetery where cypress trees released clouds of pollen. All other pollen releases occurred weeks after the main shock. Pollen clouds were seen from the stadium until San Giuliano Monastery in L’Aquila. They always appeared above the black pinewoods, which was utilized for reforestation, and were also observed from mid-April until mid-May 2009 in San Benedetto, Filetto, Bagno, Pianola, Fossa, Camarda and Poggio Picenze. Clouds of pollen dust rose up into the atmosphere following sudden gusts of wind. Pollen dust was deposited on cars and tents at the civil protection camps set up after the main shock. The clouds remained from ten minutes up to more than one hour. In some cases the clouds had an intense yellow-green color to them.

#### 3.1.2. Vegetables and Fruit

**Figure 1:**
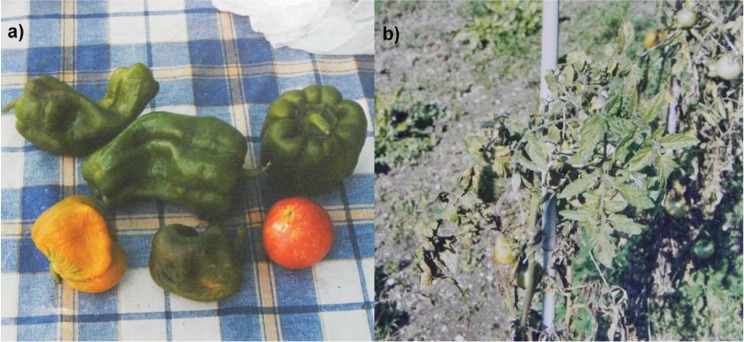
(**a**) Green and yellow bell peppers and a red tomato, from Bagno, L’Aquila, Italy, August 22, 2009; (**b**) tomatoes plants in a private garden near the railway station of L’Aquila, Italy, September 14, 2009.

Vegetable and fruit anomalies were only observed in small private gardens by their owners. The general report around L’Aquila was that fruit and vegetables had developed quickly without reaching maturity, thereby appearing ruined and wrinkled, see Figure 1. Vegetables in the photo are from Bagno, bell peppers appeared wrinkled and small while tomatoes were small with many white dots. 

This was also the case in other villages near Bagno such as Cavallette and Vallesindole. These anomalies took place in the months of August and September 2009, after the main shock. The leaves of fruit trees appeared to be burned and dried out. However, 30 km south of L’Aquila, vegetables appeared to have an exceptional development; the owners of the gardens reported that this was due to a very rainy spring and summer. This information was obtained from the villages of San Demetrio, Prata D’Ansidonia, San Nicastro and San Pio delle Camere, along Piano di Navelli. Also, the blossoming of the vegetables and fruit trees was earlier than usual, as claimed by the observer though not in accurate way. A florist in Barisciano reported that all of his shop plants had lost their petals the morning after the main shock.

#### 3.1.3. Mushrooms and Algae

Other manifestations included a dense mushroom proliferation reported between Santa Ruffina and Lucoli, covering an area of about 5 meters length and 1 meter width. The mushrooms were brown in color and were observed on April 8–9. Additionally, the water of both Lake Salto, 30 km west of L’Aquila, and Lake Scandarello, 35 km north of L’Aquila, appeared murky with reddish hints a few weeks before the main shock. Regarding the latter, other witnesses reported seeing the water colors to be in the norm.

### 3.2. Animals

A limited number of the animal behavior anomalies are reported below, classified according to species. Moreover, due to the fact that animals can share different habitats, animal species are presented in the following habitat order: ground, underground, troposphere and water. The inclusion criteria included the capacity to express precise time, place, animal type and behavior anomaly. Several people reported seeing strange animal behavior without sounds, and most people observed more than one such behavior prior to the main shock. Many people reported different animals giving birth from days up to almost one month early, as observed during other seismic events [12]. It should be remembered that, as animals die all the time, reports of isolated deaths could have been coincidences. However, they were reported here given that a correct exclusion should be considered only after having obtained a complete view of the database.

#### 3.2.1. Dogs

Nearly all of the surveyed reported hearing dogs barking immediately before all the M > 4 quakes and many before the M > 3 quakes. In many cases, the barking of dogs was heard before and after the nighttime main shock in a more or less continued way. It was not possible to verify how many cases actually began before the shock. This behavior has been reported in most of the reported historical cases [6]. Being so, reports of dog barking were included in this study only when ulterior specific observations were associated with them.

Eighty-one observations were recorded of people hearing or seeing dogs with unusual behavior before and after the many shocks reported in Table 1 of Fidani [14]. Almost all observed dogs, of all sizes, began barking up to one hour before the shocks, whereas they stopped barking immediately before the many shocks. Specifically, some small dogs acted nervously before the main shock by fleeing from their owners and by continuously entering and leaving their houses. Another dog uncharacteristically began scratching at the front door before the main shock. Moreover, several people reported that their Abruzzese shepherd dogs sought physical contact with their owners before the main shock. In other cases, dogs began barking a few minutes before the shocks, and in a few cases they barked only after the shocks. Some dogs barked and stared while others even bit their owners before the main shock. Several dogs were reported to have not been able to recognize their owners or seemed to be deaf after the main shock. The day before the main shock, several dogs refused to enter their owners’ homes. A pitbull was unusually calm while a cocker spaniel nervously ran around its doghouse in circles before the main shock. Other dogs were reported to have dug tunnels under fences before the main shock. Concerning the animal sounds, many people reported that dogs emitted sounds half way between a yelp and a howl a few minutes before the shocks. The dogs were reported to making this sound at the same point in time. Several of these dogs were reported to have had hiccups simultaneously. A few seconds later, right before the shocks, the dogs were mute and immobile. Barking, yelping and howling ceased a few second before the shocks. A dog in the city of L’Aquila died right before the main shock.

Dog nervousness was reported to begin four months before the main shock. Stray dogs, home to the shock epicenter, fled up into the mountains days before the main shock only to return after the shock. This was observed in Sassa di Preturo, something that is commonly reported in the literature [31]. During months before the main shock, many hunting dogs did not go into heat, something which usually occurs between February and March every year, as claimed by the observers, though not in an accurate way. A German Shepherd, immediately after the foreshock at 1 a.m. in Coppito, ran around its owner barking and pulling him far from his house. A small dog in Coppito urinated frequently in his owner’s house beginning about 15 days before the main shock. This had never occurred in 13 years. A four-year-old male Rottweiler in the city of L’Aquila tried to pull its owner out of her house at around 11 p.m. on April 5 while she working at her computer. In the village of Cavallette, 18 km southwest of L’Aquila, several people reported seeing dogs acting nervously, then barking for a few seconds right before the shocks. In Tussia, about 40 km south of L’Aquila, a truffle hunting dog was reported to have been barking for about ten minutes before the main shock. This dog did not have the habit of barking in this manner. In the village of San Nicastro, 40 km south of L’Aquila, dogs were reported to have been silent during the night and started barking in unison for a full minute. After this, the main shock struck. The same dogs were observed with their tails between their legs during this period.

#### 3.2.2. Wolves

Forest rangers reported seeing a wolf in a territory uncommon to wolves, near Campotosto on April 29, 2009. Also, a wolf sighting was reported around May 2, 2009, at a civil protection camp which was put under protection by forest agents. Furthermore, forest rangers reported that two packs of wolves “disappeared” from the region along the Valle Peligna. However, no exact time frame was given. Even between 458 and 16 B.C. wolf sightings near cities were considered a precise premonitory sign of earthquakes by Romans [6].

#### 3.2.3. Horses, Cows and Donkeys

Observations for 17 horses, six cows and one donkey were recorded. Horses were reported to have neighed and refused to return to their barns, before the main shock. After the main shock, they continued to neigh even louder. Horses were reported to be acting nervously before both the main shock and the strong aftershocks in Conche near Lake Scandarello, Amatrice, Montereale, Capitignano and Civitatomassa. Also, they were reported to be suffering but not neighing in Rosciolo at uncertain points in time. Horses neighed and nervously raised their front legs 30 seconds before the shocks in Lucoli.

Regarding cows, a decline in milk production of about 20–50% for weeks was reported around Cagnano Amiterno and Monticchio, after the main shock. Cows tried to break loose from their chains 4–5 minutes before the main shock in Camarda. Also, they unusually mooed 1.5–2 hours before shocks, even during the night, in the zone between Ocre and Monticchio. This incessant mooing lasted for 30-minute intervals. Many of the breeders explained the phenomenon of milk reduction as a consequence of the continuous vibration of the land that produces stress for cows. In this case, the data was recorded by the Zooprofilactic Institute of Abruzzo and Molise “G. Caporale” in Teramo, Abruzzo, Italy. Similar observations had been very commonly made during previous earthquakes in Italy [12,32,33].

Finally, a donkey was heard to bray violently seconds before the main shock during the night in Rosciolo.

#### 3.2.4. Sheep, Wild Boars and Pigs

Sheep, which were usually docile, started acting nervously weeks before the main shock up until several weeks after. Sheep began to bleat minutes before the shocks as defined in Table 1 of Fidani [14]. Bleats were reported to sound like cries of fear. On two occasions it was noted that sheep were carrying their heads high right before the shocks. Wild boars, native to Camarda, San Nicandro and Rocca Preturo, were not seen for months around the times of the earthquakes. Pigs were unusually nervously near Capitignano before the main shock.

#### 3.2.5. Hamsters, Moles, Rabbits and Rats

A pet hamster was acting nervously and scratching his paws before the shocks in Roio Piano. Many holes made by moles were observed around Roio Colle and several dead moles were seen, but at an uncertain time. A pet rabbit, living in a house in Colle Preturo, stood on his back legs and continuously sniffed the air around him for a few days before the main shock. A pet rabbit, in San Gregorio, was seen beating his legs during many shocks from December 2008. Rats were seen twice in February in Via Strinella of L’Aquila. At least three big rats were seen in the public square of Paganica village on March 30, 2009. Rats had never been seen by the witness in the center of Paganica before. Rats were found in the Regional Council Building days before the main shock. Many rats were seen in the village of Calascio after April 6.

#### 3.2.6. Cats

Many cats fled from their homes in Arischia starting in December 2008, as has been reported in previous papers [31]. Twenty-one cats fled from Sassa weeks after the main shock. Cats were missing on April 5, in the afternoon, from Poggio Picenze. These cats returned home one month later. Cats were reported fleeing about 10 minutes before the shocks in Pettino. From the morning of April 6 for some days, cats were not seen in Pettino and during the same period, a mother cat abandoned her kittens. A lady in the center of L’Aquila city had seven cats, on the evening of April 5 all her cats began to “cry”. The cats fled to the roof of the house and “cried” all together. Also, cats were reported to hide in their beds while others were seen scratching doors and windows before the main shock in Tornimparte. Cats were reported to be hyperactive and were not seen stretching from April 7–8 in Villa Sant’Angelo. A cat was said to have ran up and down floors in a house and then scratched a door wanting to escape before the main shock. Another cat remained unwilling to touch his stomach to the ground, but was willing to be held in San Demetrio on April 5. Cats in this latter area were reported to go into heat late that spring.

#### 3.2.7. Earthworms and Slugs

**Figure 2:**
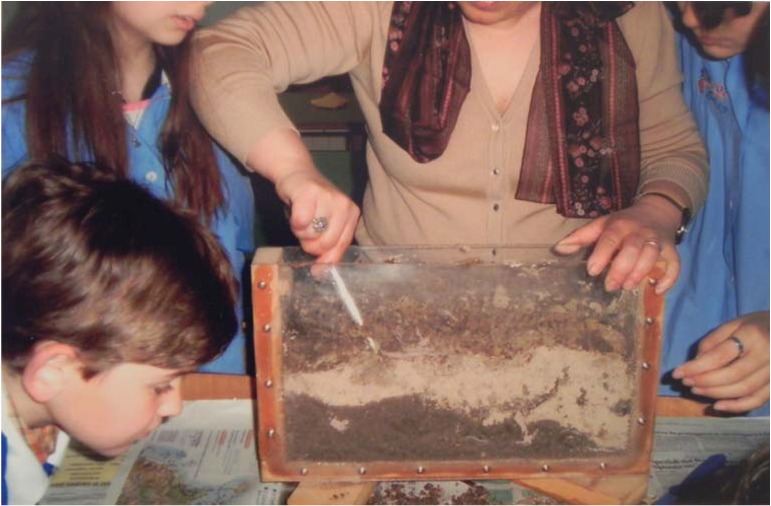
The earthworm farm at the San Francesco Primary School in Santa Vittoria in Matenano, Fermo; courtesy of Silvana Picchioni.

Before the quake, regular observations had been made at the San Francesco Primary School in the city of Santa Vittoria in Matenano, about 70 km north of L’Aquila [34], by a local teacher named Silvana Picchioni and students for a class project. This was during the academic year 2008/2009. Earthworms were kept in a semi-transparent worm farm. On the morning of April 6, at 7:50 am, a janitor entered the classroom and found the earthworms on the floor. The transparent warm box was not overturned. The janitor had visited the same classroom two days before in the afternoon and had not seen earthworms on the floor. The teacher had been keeping earthworms in an open-top box on a table for the past ten years and she reported that over these years, such an event had never happened. A photo of the box used for the experiment is shown in Figure 2. The box was constructed of two thin wood walls and two transparent plexiglass walls. The bottom of this box was made of thin wood. The box was filled with three different strata of soil: sand, peat and normal soil. Fresh earthworms were placed on the soil every September and were taken back to the wild in June every year. Up until March 17, 2009, there had been only one earthworm in the container, at this time 23 additional earthworms were placed inside the box. The soil was regularly watered every 20 days. The last time it had been watered was on March 17, 2009. The only available exit was the open face top of the box. The two transparent plexiglass walls were covered with black plastic. A total of eight earthworms were found dead. They had signs of rigor mortis. Three of these were found under a classroom window, two between the window and the table, which was positioned 5 meters away against the opposite wall. Two other earthworms were found under the same table and one was found still in the box on the table. It was reported that the classroom was usually cold during the winter months. The only radiator in this classroom was positioned under the window. The teacher referred that students were allowed to approach and touch the table. This produced many shocks, but earthworms had never tried to escape because of this.

Throughout the surveyed area, 48 people reported seeing an increased number of earthworms from January 2009 up until September 2009. The most part of these observations were made from a few days (4–6) up until a few hours before the shocks. Seldomly seen exceptionally long earthworms were seen in these situations (40–50 cm). These were unusual, as described in [35], earthworms usually still are underground during this season because of the low temperatures. A few days before the main shock, a “large” number of earthworms appeared on the streets of Cansatessa, a village 5 km north of L’Aquila. Residents said that it was almost impossible to walk at some points of the village because of the earthworm invasion. Heaps of earthworms were observed in a vegetable garden on April 5 in the village of Camarda. At the end of March 2009, dead earthworms covered a sidewalk around a house in Capitignano, about 20 km north of L’Aquila, and in the village of Montereale, before the main shock. A large number of earthworms were also seen some days before the main shock in the village of Rosciolo, about 25 km south of L’Aquila. Earthworms were also sighted near Chieti, about 60 km east of L’Aquila, the day of the main shock. They were also seen in abundant numbers in the following areas: San Sisto, Santa Barbara, Colle Sapone, Assergi, Camarda, Roio Colle, Rosciolo, Tornimparte, Cesi, Roio Piano, Santa Ruffina, the civil protection camp of Santa Vittoria, Pettino, Monticchio, Paganica, Scoppito, Coppito, Aragno, Onna, Picenze, Poggio Picenze, Barisciano, San Vittorino, Borbona, Ocre, San Demetrio and Tempera. The presence of slugs is normal in Central Italy, but they were not observed in Pettino and Colle Preturo from May to August 2009.

#### 3.2.8. Ants, Centipedes and Spiders

Near the main shock, ants appeared ahead of their season in a house in Arischia. They were reported to go under furniture in Tornimparte. Many ants were observed on the morning of April 5 in Roio Colle. Flying ants were observed in mass but disappeared suddenly in Paganica at uncertain times during the earthquakes. An anthill was seen to empty out completely with a swarm of ants completely covering a small area around the anthill in Pettino at an uncertain time. On April 6, ants formed many hills in Pettino.

Spiders were reported to have increased in numbers days before the main shock in a house in Cansatessa. The same owner reported that small ants came out of the ground many times before the shocks. Many ants were observed on the third floor of a building in L’Aquila one month before and up to the day of the main shock. Ants were seen climbing external walls of a building and their number increased steadily until April 6. Many centipedes in single files were seen on April 9 in Pettino.

#### 3.2.9. Insects

**Figure 3:**
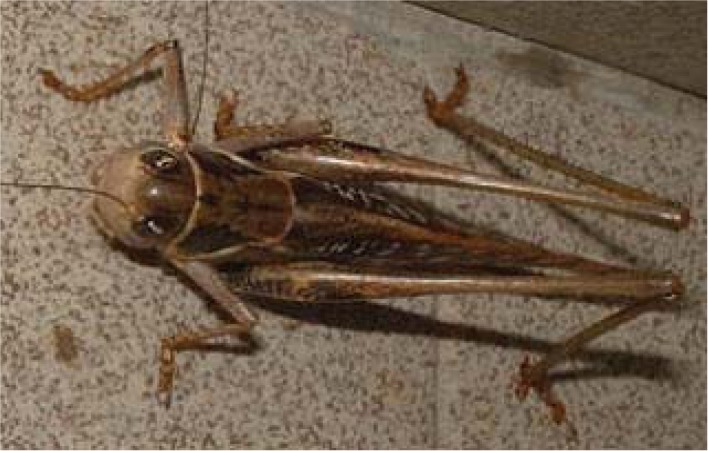
A photo of a grasshopper taken in Raiano at the beginning of August 2009.

Grasshoppers invaded the L’Aquila area three months after the main shock. It was reported to be a biblical sight on July 15, 2009. Millions of grasshoppers had invaded the territory of the epicenter starting from Bazzano and Onna and diffusing in Sant’Elia, San Gregorio, Barisciano, Poggio Picenze, Raiano, Pianola and Preturo. The insects were found in hundreds of buildings and camping sites. The health authorities disinfected the areas and the phenomenon decreased at the beginning of August. Live grasshoppers were taken and examined. Results from this analysis showed that the orthoptera were not dangerous for humans and in this case did not harm the cultivation, see Figure 3. It was determined by health authorities [36] that the grasshoppers proliferated in a recently cut grassed area near Bazzano due to the high temperature and humidity. It could be hypothesized that this proliferation was due to soil degassing which was reported to have occurred [37,38]. Grasshoppers invaded the villages of Fontecchio, Fossa, Onna and Bazzano from the city of Popoli along the Valle Peligna.

Flies native to Central Italy were reported to have lived throughout the winter inside buildings in L’Aquila. Usually these flies die by early winter, as claimed by the observer, though not accurately. Crane flies appeared on a wall in Paganica on April 8, long before fly season.

An invasion of ladybirds was reported on a snow-covered mountain of Campo Imperatore, Gran Sasso, on April 5.

#### 3.2.10. Birds

Birds suddenly stopped twittering right before an aftershock in Amatrice, 35 km north of L’Aquila. Parrots fluttered and screamed and lost feathers before shocks in Santa Vittoria. Twittering was heard on April 5 at 23:15 p.m. in Pettino. Pigeons and magpies were not seen from about March 30 in Roio Piano. Birds were not seen either in Roio Colle up until the middle of May. Two turkeys were found dead in Roio Colle after the main shock. Several dead magpies were found in the village of Santa Ruffina at uncertain times. Birds were seen to take off from treetops 10–15 seconds before many shocks. Swallows were not seen in Camarda in 2009, while many had been seen in previous years. At 1:30 am on April 6, birds were observed landing from treetops in Paganica. Birds were reported singing 30 minutes before the main shock in the center of L’Aquila. Crows were seen flying for a period of many minutes, without landing, before many shocks. Small birds were heard on many occasions “screaming” for about 10 minutes before the shocks. Pigeons and crows alighted immediately before many shocks, while other types of birds were not seen in Collebrincioni after the main shock. After the main shock, birds were heard twittering during the night in Scoppito, San Pio delle Camere and Tempera before the aftershocks.

#### 3.2.11. Hens, Geese, Owls and Turkeys

Anomalous behaviors regarding 27 hens, two geese, two owls and one turkey were reported. Hens, which were nervous some minutes before the shocks, calmed down at the moment of the shocks. Hens came out from their coop after dark before the main shock in the village of Conche of Lake Scandarello. This was unusual because hens usually do not leave their coop after dark. The same was observed in several other strong quakes [6]. Roosters crowed before the main shock and hens sang before the dogs started barking, as reported above. Hens appeared disturbed during the night of April 5–6, and roosters crowed between 3:30 and 4:00 a.m. on April 6. Hens were reported to be motionless after the main shock. Other testimonials reported hens grouped together before the shocks, as also underlined in [6]. Hens produced more eggs than usual from a week before the main shock in Colle Brincioni and Barisciano. Whereas many other hens stopped laying eggs for 1–2 weeks after the main shock. Moreover, hens were found dead with no signs of violence in Roio Colle at uncertain times. Hens and geese fluttered and “screamed” 5 seconds before the shocks. A rooster and an owl “sang” together during the night of the main shock in Santa Ruffina at an uncertain time. Hens “screamed” 3–4 minutes before the main shock and a rooster “sang” at 3:20 a.m. in Camarda. Roosters “sang” some seconds before the shocks in Paganica and Picenze at all hours. 

#### 3.2.12. Toads, Snakes and Tortoises

In the city of Chieti, about 60 km east of L’Aquila, a homeowner reported that her pet tortoises were unusually excited during the time of the shocks. In another case a tortoise in a soil box on the third floor of a house in Coppito did not go into lethargy during the winter 2008–2009. Similar observations were reported with the Kobe earthquake, M = 7.2, in 1995 [39]. Instead, it went underground every evening and came out every morning. Many snakes were observed crawling from the Vetoio River towards the parking lot of the local hospital the day after the main shock. Frogs were heard croaking before and after the main shock. Two black snakes were seen in the civil protection camp located in Pettino for days right after the main shock. Four month before the main shocks a royal python in the quarter of Torrione in L’Aquila was seen twisting. After this, it unexpectedly changed its skin and died.

Ten kilometers west of Santa Vittoria in Matenano and about 70 km north of L’Aquila a biologist working on a project regarding toad mating at the time reported that a week before the main shock the population of toads had decreased significantly on the shores of Lake San Ruffino. Interestingly, according to the biologist, given that there was a full moon on April 4–6, there should have been an increase in the number of toad matings [29]. It was interpreted as an earthquake linked phenomenon [40]. Animals came out of hibernation later in the L’Aquila region compared to neighboring regions. This is due to the lower spring temperatures in L’Aquila. Toads were not observed in the other areas of the epicenter, whereas snakes were observed in a few cases with respect to previous reports in Italy [12] and China [6].

#### 3.2.13. Fish

**Figure 4:**
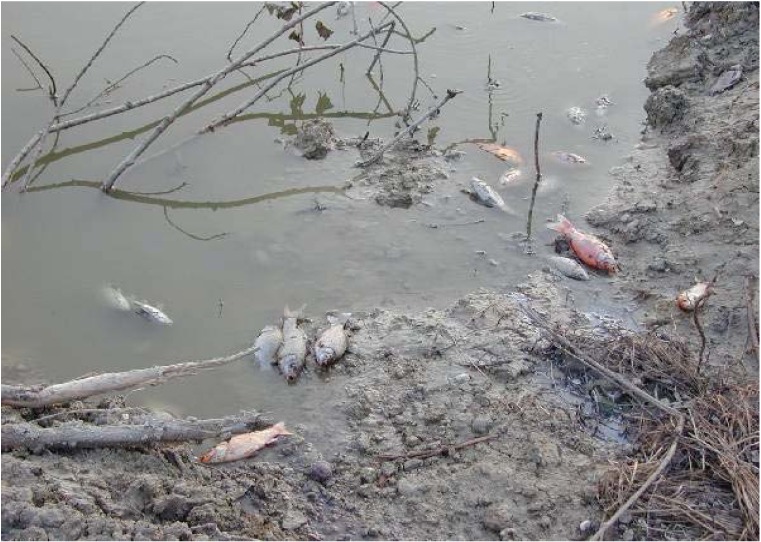
Dead fish on the surface of Lake Scanno, L’Aquila, Italy, October 10, 2008.

Many local newspapers reported that: *...nearly 220 dead fish were found on the surface of Lake Scanno* (60 km southeast from L'Aquila) *on the morning of October 10, 2008. … In particular, about 40 big whitefish and about 180 juvenile perch were found. These species had been introduced into the lake about 20 years prior*; (see Figure 4). Several local fishermen reported unusually large numbers of dead fish weeks around the seismic events. The same newspapers had stated a few months before that a fishing boat named *Goletta Verde* (http://www.legambiente.it/legambiente/about-legambiente) had analyzed the lake water finding it within the norms. Another lake water analysis carried out by ARTA (Agenzia Regionale per la Tutela dell’Ambiente) in September 2008 reported very good water conditions with levels of oxygen above average. The Zooprofilactic Institute of Abruzzo and Molise “G. Caporale” in Teramo, Abruzzo, Italy, conducted some research and on October 25, 2008 reported that: *The Institution ruled out that the fish deaths could have been related to infections. Post-mortem examinations and laboratory tests on perch did not obtain significant results; whereas those of whitefish and trout showed congestion of the peritoneum as well as excess mucus of the gills. All tests resulted negative for parasitological and bacteriological disease.* This event happened almost 6 months before the earthquake. It is noteworthy because it took place when the earthquake swarm started in L’Aquila [1]. 

Small dead whitefish and juvenile perch were sighted along the shores of Lake Campotosto, about 20 km north of L’Aquila, a few days after the main shock. In Lake Pantanis, near Piedicolle of Montereale, about 20 km northwest of L’Aquila, it was not possible to catch any carp, pike or perch from April 5 in the afternoon until some days later. A few days after, on April 10, gas bubbles were seen on the surface water of an irrigation channel near Bazzano [37,38]. Two weeks before this, local fishermen had started reporting lower catches. Another fisherman from Termine, near Cagnano Amiterno, about 20 km from L’Aquila, reported that the trout were not biting. The same observation was reported by fishermen on Lake Salto, about 30 km west of L’Aquila, the day before the main shock. About 15 dead fish, including adult carp and catfish, were found on the surface of the lake in the Prati area, south of the village Rocca di Cambio, about 15 km southeast of L’Aquila, on April 15, 2009. Dead fish were also observed along the shores of Lake Sinizzo near the village of San Demetrio, about 15 km south of L’Aquila. No dead fish were seen on the surfaces of Lake Scandarello, about 35 km north of L’Aquila, Lake Salto, about 30 km west of L’Aquila, and Lake Vetoio, about 4 km northwest from L’Aquila. The flow of the nearby springs of Lake Vetoio increased by 45% after the main shock [41].

Many cases of anomalous fish behavior were recorded, especially in the sea [42]. Especially the presence of fish, which usually are not found in that area, and also the disappearance of fish was observed [43].

### 3.3. Humans

Humans’ sensations had previously been collected by questionnaires in Italy and reported for the Piedmont earthquake, M = 5.7, of 1808 [33], Liguria earthquake, M = 6.5, of 1887 [10], Friuli earthquake, M = 6.7, of 1976 [6,12,44], Irpinia earthquake, M = 6.9, of 1980 [12], in China, Liyang earthquake, M = 6, of 1979 [45], in Rumania, Vrancea earthquake, M = 6.9, of 1986 [46] and in Japan, Kobe earthquake, M = 7.2, of 1995 [47]. The abundant material collected in these cases motivated the section concerning human sensation for the questionnaire used here [14]. It should be remembered that most of the anomalous disturbances reported were not medically diagnosed, and for this their validity could be doubted. Here, 178 human health disturbances were collected. These included: dizziness, thermal variations, anxiety, tiredness, skin rash, insomnia, nervousness, respiratory difficulties, vomiting, headache, tachycardia, dried lips, nausea, allergies, herpes, focal disturbances, conjunctivitis, nose bleeding, swelling of lips, blisters and even death. Panic attacks, jerky vision, nausea and dizziness were reported by persons outdoors at the time preceding the stronger shocks in different locations. These health disturbances were particularly intense on the morning of April 4 and the evening of April 5, in Coppito.

#### 3.3.1. Dizziness, Nausea, Vomiting and Tachycardia

Thirty-nine reports of dizziness and nine reports of vomiting were collected. Two girls, one from Santa Vittoria and the other from Verrico, reported having bouts of vomiting and dizziness from the beginning of March up until April 6. Many girls from a secondary school in L’Aquila also reported vomiting and dizziness over the same period. Dizziness was reported to begin in December 2008 for several people in Cansatessa and Centicolella. While on March 30, many people fell sick with dizziness in Cagnano Amiterno, Rosciolo and Sassa. One person experienced dizziness in her office, which was on the ground floor of a building in Via Strinella of L’Aquila, starting from December 2008 and lasting up until the quake. She had never experienced such dizziness before. Her colleagues also experienced the same dizziness. Dizziness and tachycardia were reported in the quarter of Torrione in L’Aquila on April 6 and the following days. Another person referred to being dizzy in the evening of April 5 at home in his fourth floor apartment in the same quarter. Dizziness was reported in Vallesindole starting from December 2008. Several people from this village remembered that they had felt continuously dizzy, independent of location in the village. Dizziness was reported in Fossa starting from December to January, and more intensely from February to March, in every location and at all hours. In L’Aquila, a person reported being regularly dizzy from December 2008 up until April 6, 2009, when this dizziness disappeared. A lady had a tachycardia attack in the evening of April 5 in San Marco di Preturo. Dizziness and nausea were also reported in 10% of the population for Friuli and Irpinia earthquakes [12].

#### 3.3.2. Thermal Variations

Twenty cases of abrupt thermal variations were recorded during the shocks. Most cases were characterized by hot flashes during the shock and cold flashes afterwards. These testimonies came from people living in L’Aquila, Capitignano, Civitatomassa, Pagliare di Sassa as well as 14 other villages. A man from Paganica, one of the villages, reported feeling hot right before the main shock, and during it he felt as if he had been enveloped in fire. Several health care professionals at the local hospital on March 30 complained of hot foot soles.

#### 3.3.3. Skin Rashes and Tingling

Several cases of skin rash were reported at the civil protection camps after the main shock. Rash was reported by several people in Pizzoli after the main shock. A man felt a tingling under his feet for two days after the main shock in Camarda. Many people reported chapped lips and dry skin after the main shock in Camarda. A lady living in Bazzano reported skin irritation, characterized by spots and itching, on her forehead starting from December 2008. She had never had such a problem before. Frequent sneezing was reported by several people living in L’Aquila from about three months before the main shock and ending after the main shock. Many people suffered from chapped lips starting from April 6 for about 20 days in Onna, Tempera and San Biagio. Several people in Onna reported having herpes and swelling of the upper lip for 20 days after April 6. A pharmacy reported that there had been an increase in the request for antifungal medications, lice creams, especially for men and school children, and products for skin allergies at uncertain times. While requests for herpes medications remained in the norm.

#### 3.3.4. Anxiety, Nervousness, Insomnia, Tiredness and Headache

Unexplained feelings of anxiety were reported by several people in Pizzoli for some months up until the main shock. Fatigue and insomnia were reported before the main shock in Pizzoli by a man. One person complained of nervousness and insomnia from 23:30 p.m. on April 5 in Tornimparte. Fatigue was reported by a person starting from about two weeks before the main shock. More people reported intense sleep right before the stronger shocks in the civil protection camps. This effect was also recorded by a person living in a third floor apartment during the March 30, M = 4, shock. All of these cases were reported to have lasted only a few seconds. Respiratory difficulties and anxiety were reported on the evening before the main shock in Civitatomassa, characterized by a feeling of heaviness, accompanied by tremors. A headache was reported by a person in Preturo before and after the main shock who had never suffered from headache before. The same feelings were reported with an occurrence of up to 30% of the population in the occasions of the Friuli and Irpinia earthquakes [12].

### 3.4. Type-Time and Type-Space Distributions

The first step of this study began by ordering and classifying the reports. This was done in the previous paragraphs by associating types with similar characteristics for each category of living organisms. Then it was possible to temporally order the reports with respect to the times of the shocks, see Table 1 of Fidani [14]. A special focus was placed on the April 6, 2009 main shock, in order to determine what happened before, during and after. When it was not possible to define the order, it was called uncertain. The order was determined by comparing the shock times with the beginning times of anomalous behavior, even if the latter had lasted for a long time. This order is summarized in Table 2, Table 3, Table 4, Table 5, where the total number as well as a more accurate type description are reported on plants, animals, humans and all the cases, respectively.

**Table 2:** Plant summary.

Plants	Before	After	During	Uncertain	Total
Pollen	1	18	1	-	20
Flower	-	-	-	1	1
Grass	-	-	-	1	1
Tree	-	2	1	1	4
Fruit	-	7	-	-	7
Vegetable	-	12	-	-	12
Algae	3	-	-	-	3
Mushroom	-	2	-	-	2
Sum	4	41	2	3	50

**Table 3:** Animal summary.

Animals	Before	After	During	Uncertain	Total
Dog	63	2	-	16	81
Cat	17	3	1	8	29
Horse	9	1	1	6	17
Cow	4	2	-	1	7
Sheep	4	-	-	1	5
Wolf	-	-	-	4	4
Donkey	-	-	1	-	1
Wild boar	-	1	-	2	3
Pig	-	-	-	1	1
Rabbit	3	-	1	-	4
Mope	1	-	-	1	2
Goat	-	-	-	1	1
Hamster	1	-	-	-	1
Rat	3	3	-	-	6
Earthworm	25	10	-	13	48
Slug	-	-	-	2	2
Snake	1	3	-	3	7
Toad	1	1	-	1	3
Turtle	-	1	-	1	2
Fish	4	-	-	5	9
Bird	16	5	-	10	31
Parrot	4	-	-	-	4
Hen-roost	19	1	1	6	27
Geese	1	-	-	1	2
Owl	1	1	-	-	2
Turkey	-	1	-	-	1
Grasshopper	1	4	-	-	5
Bee	-	-	-	2	2
Ladybug	1	-	-	-	1
Flies	1	-	-	-	1
Crane flies	-	1	-	-	1
Ant	5	1	-	3	9
Centipede	1	-	-	1	2
Spider	1	-	-	1	2
Sum	187	41	5	90	323

**Table 4:** Human summary.

Human	Before	After	During	Uncertain	Total
Dizziness	19	7	1	8	35
Nausea	4	4	-	1	9
Vomit	7	1	1	-	9
Anxiety	7	1	2	1	11
Nervousness	6	1	-	1	8
Tiredness	4	1	-	2	7
Thermal	7	1	9	3	20
Rush	2	3	-	5	10
Dry skin	-	7	-	-	7
Erythema	1	2	-	1	4
Herpes	1	2	-	3	6
Allergy	1	-	-	1	2
Tingling	-	1	-	2	3
Tachycardia	2	1	-	-	3
Headache	9	1	-	2	12
Respiratory disturb	6	-	-	-	6
Heaviness	2	-	-	-	2
Tremors	-	1	-	-	1
Dry lip	-	10	-	-	10
Insomnia	5	-	-	-	5
Ocular disturb	1	1	-	1	3
Swelling	-	1	-	-	1
Blob	1	1	-	-	2
Blister	1	-	-	-	1
Dead	1	-	-	-	1
Sum	87	47	13	31	178

**Table 5:** Biology summary.

Biology	Before	After	During	Uncertain	Total
Sum	278	129	20	124	551

It needs to be stressed that many people affirmed that their reports regarding dogs, cats and vegetables, lacked a precise quantity. The multiple reports, including the ones mentioned above, when not explicitly distinct, were considered as only one case. The same thing was valid for human disturbances. In each camp people live together so they can discuss their experience of a particular disturbance. This can produce bias, and for this reason, experiences were reported which were experienced in first person. Human multiple reports as well as pharmacy reports, when not explicitly distinct, were also considered as one case. Being so, a part of the cases reported in the tables were grouped to avoid the influence of communication.

**Figure 5:**
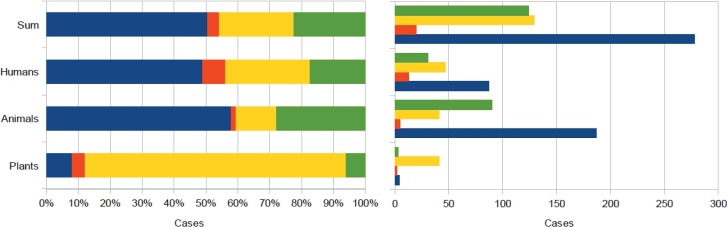
A summary of relative and absolute occurrence of time ordered observations with respect to the biological types, based on Table 2, Table 3, Table 4, Table 5.

**Figure 6:**
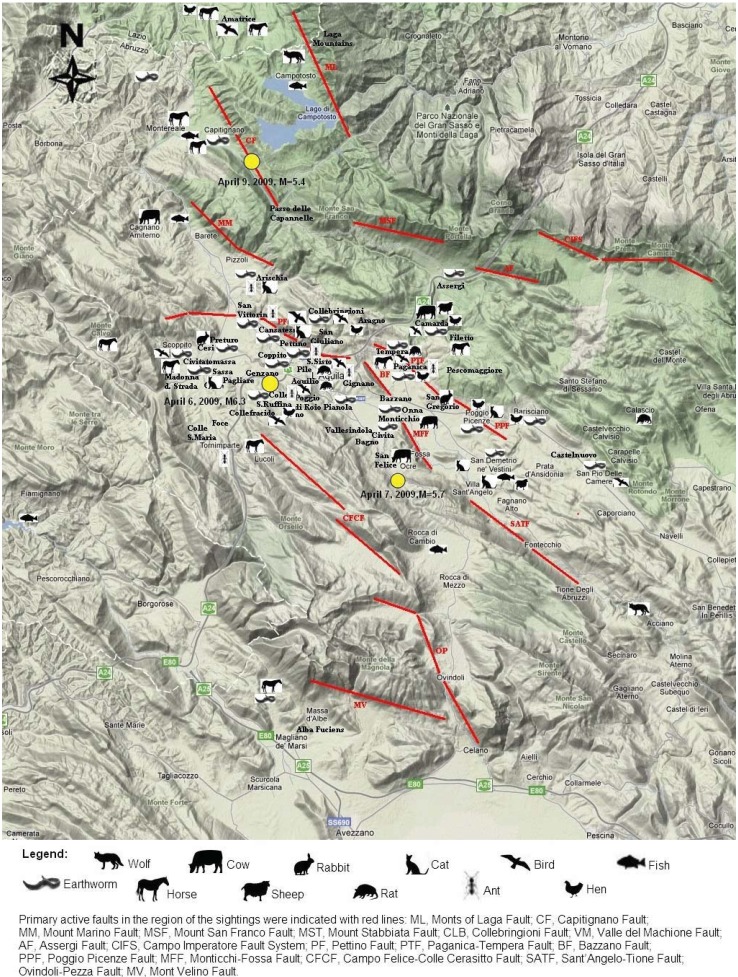
The geographical distribution of strange animal behavior with the legend; yellow circles indicate the stronger shocks of the activated seismic sources.

Figure 5 reports data on the relative and absolute occurrence of time ordered observations with respect to the biological types from Table 2, Table 3, Table 4, Table 5. The principal result of this schematic summary was that anomalous behavior occurred mainly before shocks in at least half of the cases. This trend is similar to that of the human subgroup. Anomalous behavior for the animal subgroup was almost 60%. Animal reports constituted nearly 60% of the total database and are reported in a geographical distribution in Figure 6. It should be remembered that information regarding dogs was only partially reported, this was only when something more than simple dog barking was observed. Based on this decision, and to avoid the overcrowding of Figure 6, dogs are not reported in the geographical distribution. Regarding anomalous behavior occurring principally after the shocks for plant subgroup see Figure 5. This summary suggests that the influence on flora was either not evident or delayed. Indeed, these observations were few in number.

The geographical distribution of anomalous animal behavior is represented on a map, as was done for earthquake lights [14,16]. A series of symbols was used to display some of the animals identified in the previous paragraphs above. This is shown in Figure 6 only for those reports regarding anomalous animal behavior before the shocks, excluding dogs. Several reports were made in places that are not included on the map. Observations made out of the Region of Abruzzo including those more than 40 km from L’Aquila were not uniformly distributed around the epicenter. Being so, they were not considered for a geographical discussion of biological anomalies. A geographical distribution of principal active faults is also indicated by red segments, while the positions of the three principal seismic events of April 2009 are indicated by yellow circles.

### 3.5. Discussion

Figure 5 clearly indicates the prevalence of anomalous animal observations before the earthquakes. However, it must be remembered that times before strong quakes were seldom described as being very calm [6], so it might be difficult to highlight premonitory anomalies. Furthermore, human observational attention is not the same before and after a strong earthquake, which can disrupt a person’s life [48]. Finally, strong earthquakes can also modify animal behavior for a long time [6], changing their habits. These three considerations must be taken into account in order to reduce their influences. This could be done by introducing a further section in the questionnaires. The latter two considerations can explain the relative difference between human and animal anomalous observations following quakes. Regarding the second consideration, humans can be disoriented regarding their feelings for long time. Whereas anomalous animal behavior becomes difficult to distinguish due to the third consideration. These two considerations may account for the opposite relative difference between human and animal anomalous observations at uncertain times, see Figure 5. Observations during the quakes were less than 10% for the three groups. However, reports were more numerous for humans, due to thermal sensations, whereas they were very low, near 1%, for animals. Perhaps the latter was due to the earthquake roar that overwhelmed animal sounds, as well as a lack of attention on the part of witnesses during the shock. 

Reports dealing with plants occurred principally several weeks after the main shock of April 6, 2009 and other strong earthquakes of the sequence, while a few cases occurred before the main shock and were observed far from the epicenter. Case histories have reported some significant observations for the Friuli earthquake of May 6, 1976 [12], M = 6.7, which occurred in the same season of the L’Aquila earthquake, as well as some Chinese [6,47] and Japanese [47,49] earthquakes. In all these cases, similar characteristics of fast plant growth and an abundant presence of yellow flies were observed together with abundant rainfall and fast soil drying [12], which was also observed in L’Aquila [50]. Electric field and electromagnetic impulses produced experimental effects on plants consistent with past observations before earthquakes [47,51]. Pollen was the most frequent observation made around L’Aquila, where the survey was carried out. However, Forest Rangers reported that yellow-green clouds were always very common in this region during May, so it is not possible to establish if they were quantitatively exceptional without instrumental measurements during the aftershock period. Moreover, meteorological fluctuations can also strongly affect the pollen release. It was more probable that these observations were made as people were living in outdoor living arrangements, which made them more aware of their surroundings. Fruit and vegetable reports in August and September 2009 were non-uniform regarding plant response in the epicenter territory. Plants in all the L’Aquila province appeared to generally develop faster than usual in the spring and at the beginning of the summer, as claimed by the observers, though not accurately. However, they failed to mature fully in a restricted area in L’Aquila and within a 5 km radius around it, in Aquilio, Tempera, Bagno and Onna for example. Whereas plants reached good maturation, with very good harvests, south of L’Aquila and more than 20 km from the city, in San Demetrio, Barisciano, San Pio delle Camere and San Nicastro. Being so, a particular behavior of exalting maturation at a certain distance from the epicenter and impending maturation in the epicenter could be observed. However, unknown local variations in rainfall can be important, and the number of the reported cases is not sufficient to establish this distribution with certainty. Furthermore, a better-controlled history of these plants would be necessary to reveal fungi or other parasite attacks, which are determinant for their maturation [52]. The observations suggested algae proliferation three times on two lakes near L’Aquila. These were the only observable effect of plants occurring before the main shock. However, they were observed on only two lakes where dead fish were not observed. The presence of algae was usually linked to a lack of oxygen and, therefore, to dead fish [53].

Dead fish were also found in other Abruzzo lakes from 15 to 60 km around the epicenter, north and south of L’Aquila. Non-biting fish were reported principally only north of L’Aquila. CO_2_ measurements revealed slightly rich springs after the main shock [37,54], and considering the observed dead fish and lake bubbles [38], it could be a confirmation that degassing activity had already been activated months before April 6, 2009 [55]. Moreover, in December 2008, at the beginning of the seismic swarm, dead fish were observed in Lake Scanno, 60 km southeast of L’Aquila, and possibly this was not due to water pollution. Strange toad behavior [29] was also observed on Lake San Ruffino, 70 km north of L’Aquila, and is thought to have been due to the abnormal formation of hydrogen peroxide in the lake water [40], following the hole emission process [56]. Far observations, >20 km, also occurred for lake algae, although this and the dead fish phenomena regarded different lakes and hence cannot be causally connected. Another observation far from the epicenter concerned tortoises. Hence, most of the far observations occurred in or near water.

A suggestion for an electrical link to anomalous behavior in fish and other aquatic animals before earthquakes was given [6]. An exception regarded the case of dead earthworms found at a school in Santa Vittoria in Matenato, which is also near Lake San Ruffino, 10 km away. It cannot be due to seismic waves because of the far distance from the epicenter (70 km). However, gases or electrical currents could not have acted directly on this box either. Considering this, the Santa Vittoria in Matenano observation remains unexplained. Earthworm observations were one of the more significant animal behaviors noted for their early response to the seismic shocks, as reported in Table 2. Other than the relevant number of sightings, their observation times (hours–6gg before the shocks) were earlier compared to those for cats, dogs, horses, birds and hens, which occurred up to hours before. Earthworm observations had been reported for past earthquakes [15,47,57] with similar anticipation times to those observed for L’Aquila. A possible influence of electric or electromagnetic ultra-low-frequency waves on these animals has been experimentally shown [47].

Most animal reports concerned domestic animal observations, and their early observation times lasted from a few seconds up to a few hours. This has been reported in past works [57], where the shift of precursor time towards smaller values regarding observations of larger animals was evidenced. Electrical discharge experiments were made using a Van de Graaff generator in the presence of dogs and cats [58]. These showed that dogs avoided the discharge source and barked, while a cat wandered away meowing lugubriously taking with it its owner’s socks. Declines in milk production and cow dancing have also been reported to occur on some farms where electric and magnetic fields (60 Hz) are not well shielded and simultaneously introduce harmonics [59]. In fact, milk decreased as the number of 3rd, 5th, 7th, 21st, 28th, and 42nd harmonics increased, and harmonics were correlated with the number of electrical line transients. After installing the shielded neutral isolation transformer, cows stopped dancing and milk production gradually recovered to its highest previous level after about 18 months [59]. Similar studies have confirmed that exposure (10 kV m^−1^, 30 μT, 60 Hz) resulted in moderate (5%) decreases in both milk yield and milk fat percentage as well as an increase in dry matter intake in non-lactating non-pregnant dairy cows [60].

Regarding spatial distributions for animals, the sightings were made principally along the river Aterno and the respective valley, see Figure 6. In fact, contrary to earthquake lights [14], which were visible from a long distance during the night, animals had to be observed from a near position to reveal strange behaviors. Being so, they were reported only by people living and working mostly along the Aterno Valley and concentrated in the epicenter. It must be remembered that the collection was geographically not uniformly distributed, being that it was concentrated in the civil protection camps and along the communication routes. Even if civil protection camps held people from many locations, a significant level of non-uniformity remained. On the other hand, animals were also non-uniformly distributed among natural parks, cultivated plains and populated regions of the epicenter. Specifically, unusual wolf observations were made outside of L’Aquila. These might have been due to the natural wolf habit to avoid populated regions. On the contrary, ants were only observed in the epicenter region, whereas horses and earthworms were observed in all of the examined territory. To have a better monitoring of unusual animal behavior distribution in future surveys, a more uniform exploration of the territory should be conducted and questions regarding natural character of any region need to be included.

Human skin diseases were reported after the main shocks after people were transferred to the civil protection camps. Being so, they were exposed to sunlight and atmospheric agents for a long time, which could have been the cause of skin problems. The feelings of hot and cold were reported by many during the shocks and a few moments before. This suggests that there might be a direct effect of earthquake energy during the shocks, perhaps microwave or visible electromagnetic waves, which is in agreement with previous reports on contemporary lights [14]. Anxiety and nervousness, as in the Friuli earthquake [6], was reported minutes before the shocks in several cases, suggesting the presence of positive hole emissions in the air [56], which are indicated to be responsible for serotonin modulation in humans and other mammals [6,61]. Headaches were reproduced during discharge experiments using a Van de Graaff generator [47]. Whereas respiratory difficulties could be produced by aerosols and CO_2_ soil degassing [6], degassing of CO_2_ and other gases from the ground [54], something which had already been revealed in the post degassing phase of the L’Aquila earthquake [37,38,54]. About 10 km eastward from Santa Vittoria in Matenano, near the village of San Procolo, in Fermo, 70 km from L’Aquila, there was an operative seat of the Central Italy Electromagnetic Network (CIEN), which has been monitoring the electric field continuously since 2006 [26]. This CIEN station recorded electric signals that were estimated to be in accordance with those produced by atmospheric electric charges [34]. 

## 4. Conclusions

A questionnaire was prepared to collect testimonies regarding biological and other non-common phenomena from Abruzzo residents after the strong quake of April 6, 2009. In a more recent re-exam of questionnaires, one thousand and three hundred phenomena were reported, 551 of which were biological phenomena. Several photos included subjects of anomalies. At least 278 of such phenomena occurred before the main shock and other strong events of the seismic sequence. Many of the domestic animals were reported to have a remarkable capacity at anticipating the stronger shocks, even up to hours before. Earthworms and ants were reported to behave strangely several days before the quakes. These results are in agreement with past observations regarding anomalous animal behavior around strong seismic events, where shifts of precursor times towards smaller values regarding observations of larger animals [57] were confirmed.

The aim of this study was to give an order to the biological observations, halfway between collecting and reproducing experiments, which is the necessary intermediate scientific step for preparing the data for a statistical study. The habitat order was chosen to associate animal species: ground, underground, troposphere and water. This order permitted to separate animals by their physical dimensions. Furthermore, this order evidenced that far observations occurred principally in and near water. Based on the spatial distribution observation (Figure 6), this work would have produced more reliable results if it had had a better sampling of observations. In the future, sampling should include a larger number and more uniform distribution of samples to avoid territory influences on the collection, as also suggested by the statistical analysis of the earthquake light collection [16]. The grouping of observations allows preparing the collection to test the statistical distributions [62], after evidencing case by case all the precise measures of space and time. Moreover, more objective criteria are also required in future studies to collect optimal data, including documented information on the case history of plants, a database of habitual animal behavior, farm surveillance as well as retrospective studies on human health issues. Three further investigation considerations should be introduced into future questionnaires to reduce their influence on data collection: (1) different perceptions of the land before and after strong quakes, considering that early times are seldom described as very calm, therein highlighting premonitory anomalies; (2) human observational attention is not the same before and after a strong earthquake; (3) strong earthquakes can modify animal behavior for a long time, changing their habits.

Human skin diseases after to the quake, especially in those living in outdoor arrangements such as the civil protection camps, could have been caused by overexposure to sunlight and atmospheric elements. This should be remembered for future events to protect the human population. Three different physical phenomena could have been the causes of sensations experienced by people over the three time intervals before the earthquakes. It is plausible that dizziness and vomiting weeks and months before the main shock were due to CO_2_ emissions, probably linked to degassing activity, whereas hole emission could have produced anxiety and nervousness minutes and hours before the main shock. Finally, electromagnetic waves could have produced hot and cold sensations seconds before and during the main shock. Several observations were collected that do not support these conclusions. Experiments reported by past authors [6,34,47,56,57] and discussed above seem to sustain two hypotheses regarding the observations from the L’Aquila Region. One regarding the presence of electric fields and the other concerning the presence of charged aerosols, both conditioned by degassing activity in the same region. Furthermore, the experiment at the San Francesco school in Santa Vittoria in Matenano—because of its mysterious nature—leads to the suggestion that placing earthworm farms in schools may help in educating children on scientific methods.
